# Mildly elevated diastolic blood pressure increases subsequent risk of breast cancer in postmenopausal women in the Health Examinees-Gem study

**DOI:** 10.1038/s41598-022-19705-4

**Published:** 2022-09-26

**Authors:** Katherine De la Torre, Woo-Kyoung Shin, Dan Huang, Hwi-Won Lee, Aesun Shin, Jong-koo Lee, Hae-Young Lee, Daehee Kang

**Affiliations:** 1 Department of Preventive Medicine, Seoul National University College of Medicine, Seoul, 03080 Korea; 2 Department of Biomedical Sciences, Seoul National University Graduate School, Seoul, 03080 Korea; 3 Cancer Research Institute, Seoul National University, Seoul, 03080 Korea; 4 Department of Family Medicine, Seoul National University Hospital, Seoul, 03080 Korea; 5 Department of Internal Medicine, Division of Cardiology, Seoul National University Hospital, Seoul, 03080 Korea; 6 Department of Internal Medicine, Seoul National University College of Medicine, Seoul, 03080 Korea; 7 Integrated Major in Innovative Medical Science, Seoul National University Graduate School, Seoul, 03080 Korea

**Keywords:** Risk factors, Cancer, Predictive markers, Breast cancer, Cancer epidemiology, Cancer prevention

## Abstract

Epidemiological evidence suggests that hypertension is associated with breast cancer risk. However, previous studies disregard blood pressure components in the healthy population. We aimed to examine the relationship between systolic and diastolic blood pressure and breast cancer risk in a Korean population-based prospective cohort. A total of 73,031 women from the Health Examinees Gem Study were followed from baseline (2004 to 2013) through 2018. Systolic and diastolic blood pressure were measured by trainee physicians at baseline recruitment and then categorized based on the international guidelines for clinical hypertension. Associations between systolic and diastolic blood pressure with overall breast cancer and stratified by premenopausal and postmenopausal status were evaluated using adjusted multivariable Cox proportional hazard regression. A total of 858 breast cancer cases were recorded for a median follow-up period of 9 years. Compared with the normal DBP category (< 85 mmHg), the normal-high category was positively associated with breast cancer risk in postmenopausal women (85–89 mmHg, HR 1.73 95% CI 1.28–2.33), but not in premenopausal women (85–89 mmHg, HR 0.87 95% CI 0.56–1.35). Similar results were found when all cases of self-reported hypertension were excluded. Results for SBP did not show a significant association with breast cancer risk. The association between DBP and breast cancer suggests DBP could be an important factor in cancer prevention, especially for women after menopause. Our study provides a first detailed approach to understanding the importance of diastolic blood pressure for breast cancer prevention and warrants further investigation.

## Introduction

Hypertension and breast cancer (BC) are two of the most prevalent chronic diseases globally^1,2^. Hypertension worldwide prevalence is around 1.3 billion^3^, and more than 2 million new BC cases are diagnosed each year worldwide. Approximately, 45.4% of the BC incidence correspond to Asia^2^. Both diseases are caused by multifactorial environmental and genetic factors, proposing an intricate relationship between hypertension and breast cancer incidence risk.

Two recent meta-analyses have reported that women with hypertension had 7% to 15% higher risk of developing BC. However, BC risk has been shown to be greater in postmenopausal women than in premenopausal women, explained mainly by hormonal influence^4,5^.

Despite the evidence of the positive association of hypertension and BC risk, few studies have reported a relationship between blood pressure (BP) measurements, systolic and diastolic blood pressure, and BC risk. A large prospective European study found an increase of 3% of BC risk per each 10 mmHg increase in systolic blood pressure (SBP) and diastolic blood pressure (DBP). Additionally, a categorized SBP and DBP by American and European hypertension guidelines have also demonstrated a positive association with BC risk^6^. In the Women’s Health Initiative cohort study, DBP over 85 mmHg was associated with increased risk of total BC (1.55; 95% CI 1.02–2.36) in postmenopausal women^7^. Conversely, European and Australian prospective cohort studies found no association between continuous or categorized BP measurements and BC risk^8,9^. Only two Asian retrospective cohort studies, in Japan and Taiwan, have studied the association between BP and BC risk^10,11^. These studies mainly focused on BP as a combination of systolic and diastolic measurements but did not evaluate them individually^8–10^. Since SBP and DBP physiopathology differs, more research is needed to clarify the association between BP components and BC risk. Furthermore, to the best of our knowledge, there is no study relating SBP or DBP with BC risk in an Asian population, where both hypertension and BC prevalence are trending upward^2,3^. Thus, this prospective study aimed to investigate the association between systolic and diastolic blood pressure and BC risk by menopausal status in a large prospective cohort of Korean women.

## Materials and methods

### Study population

Health Examinees (HEXA) study is a large-scale genomic prospective cohort study. The study population includes 173,202 participants from 38 health examination centers across Korea from 2004 to 2013. Detailed information on the HEXA study has been described elsewhere^12,13^. Briefly, information on socio-demographic and lifestyle characteristics, medical history, and diet habits was collected from a structured interview-based questionnaire, and a physical examination was performed by trained medical staff at recruitment. All participants provided written informed consent before enrollment and were followed up according to a standardized study protocol.

**Figure 1:**
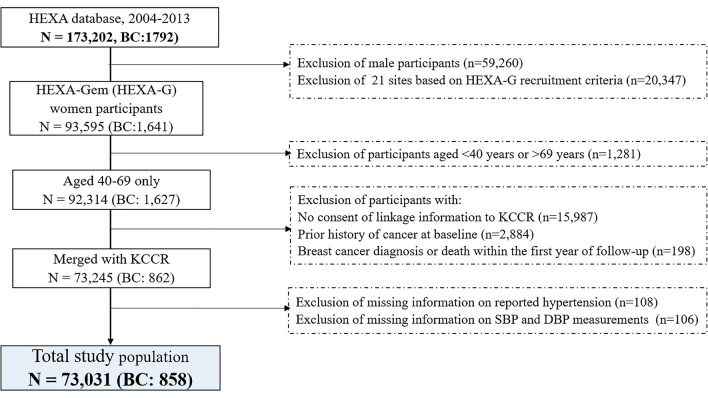
Flow chart of selection of study participants. *KCCR* Korea Central Cancer Registry, *SBP* systolic blood pressure, *DBP* diastolic blood pressure.

This study used the Health Examinees-Gem (HEXA-G) sample which comprised women from 17 participating sites with additional exclusion criteria: (1) sites that participated in the pilot study only; (2) sites that did not meet the HEXA standards for biospecimen quality control; and, (3) sites with less than two years of participation in the study^14^. Among 92,314 women aged 40 to 69 years old included in the HEXA-G study, those without consent of linkage information to the Korea Central Cancer Registry (KCCR) (n = 15,987), those who had a prior history of cancer before enrollment (n = 2884), and those who were diagnosed with or died of BC within the first year of follow-up (n = 198) were excluded from the study. Additionally, participants who had missing information on self-reported hypertension (n = 108) and missing information on SBP or DBP measurement (n = 106) were also excluded. After exclusion criteria, 73,031 women remained in the study (Fig. 1).

### Blood pressure assessment

Systolic blood pressure (SBP) and diastolic blood pressure (DBP) measurements were taken in a sitting position at least twice with a one-minute interval from both the right and left arms using a standard-calibrated mercury sphygmomanometer by trained staff during the baseline examination. If the BP in the same arm differed by more than 5 mmHg, the measurement was repeated until the last two BP values were similar. If the BP difference between the left and right arms was greater than 10 mmHg, BP was re-measured using the same procedure. If the BP was elevated after five minutes of rest, the measurement was repeated, and the lowest value was considered the most accurate. The average of the SBP and DBP measurements were recorded. We categorized SBP and DBP based on the International Society of Hypertension (ISH) guidelines^15^. BP values were classified in 4 categories, < 130, 130 to 139, 140 to 159 and ≥ 160 mmHg, for SBP; and DBP were categorized as < 85, 85 to 89, 90 to 99, and ≥ 100 mmHg. Additionally, SBP and DBP were binary categorized (SBP; < 130, ≥ 130 mmHg; DBP; < 85, ≥ 85 mmHg).

Self-reported hypertension data were collected by in-person interviews. Hypertension was assessed through responses to two independent questions, “Have you ever been diagnosed with hypertension by a physician?” and “What is your current hypertension treatment status?”. Individuals who answered “yes” to the first question and/or “currently on treatment” in the second question were considered to have self-reported hypertension. Although 12,298 participants had a previous diagnosis of hypertension, information on specific types of hypertension treatment, including antihypertensive drug use was unapproachable due to highly missed responses.

### Breast cancer ascertainment

The primary outcome was defined as the first occurrence of BC of an International Classification of Disease, 10th Revision (ICD-O) C50 code. BC cases were identified through the Korea Central Cancer Registry Data by a unique Korean resident registration number, and then merged with the HEXA-G dataset.

### Covariates

Covariates were selected based on previously published evidence of their relationship with BC risk. Demographic information including age at enrollment, education (≤ middle school, high school or College; bachelor or higher), self-reported lifestyle and health-related factors including smoking status (never, ever), alcohol consumption (never, ever) were obtained. Physical activity variable was divided into regular sweat-inducing exercisers and no regular exercisers. Weight and height were measured using standard protocols at baseline recruitment, and body mass index (BMI) was calculated as weight divided by the squared of the height (Kg/m^2^). Other covariates included family history of BC, family history of hypertension diagnosis among first-degree relatives, and reproductive factors. Proportional split was used for variable categorization for reproductive numerical variables like age at menarche (14 years or lower, 15 years, 16 years or more), age at first pregnancy (< 25 years, ≥ 25 years, no pregnancy), breastfeeding for at least one child (yes, no). Women were considered postmenopausal if they reported not having a menstrual period over the past 12 months. Women who did not provide information about their menstrual status were considered postmenopausal if they were over 55 years old consistent with previous literature for Korean women^16^. Additionally, for postmenopausal women, the use (never, ever) of hormone replacement therapy (HRT) were included.

### Statistical analyses

Baseline characteristics were summarized according to binary SBP and DBP using means and standard deviation (SD) for continuous variables and relative frequencies for categorical variables. Differences between continuous variables were analyzed using the student t-test, while chi-squared test was used for categorical variables. Cox proportional hazard regression models were used to estimated hazard ratios (HR) and 95% confidence intervals (CI) for breast cancer risk associated with SBP and DBP. Age at follow-up was used as a time scale accounting for left truncation of age. Entry time was the age at BP measurement at baseline. Exit time was the age at first incidence breast cancer diagnosis, date at death, loss of follow-up, time of censoring, or end of the study period (December 31, 2018), whichever occurred first.

For the main analyses, we performed Cox proportional hazard regression analysis to describe the BC risk for every increase of 10 mmHg. We also examined the hazard for BC by SBP and DBP categorized groups based on the ISH. Categories < 130 mmHg and < 85 mmHg were the reference group for SBP and DBP analyses, respectively.

Finally, to allow comparison with previous studies, self-reported hypertension was defined as a binary (yes/no) variable, and assessed as exposure with BC risk. Linear trends across SBP and DBP categories were tested, assigning each category's median value and then analyzing the categories as continuous variables within the model.

The Goodness of fit (GOF) test was used to assess proportional hazard assumptions for all variables included in the models, based on the Schoenfeld residuals. The multivariate models were adjusted for family history of breast cancer, BMI, age at birth of a first child, age at menarche, breastfeeding, physical activity, alcohol consumption, smoking status, and self-reported hypertension. Hormone therapy use was adjusted in all models except in the premenopausal group. Following, we evaluated whether the association varied by BMI categories (< 23, 23 to < 25, ≥ 25 kg/m^2^), age at baseline (40 to 49, 50 to 59, and 60 to 69), family history of hypertension (yes/no), history of cardiovascular diseases (CVD), and physical activity. Interaction tests were calculated using Wald test of cross-product terms.

We performed sensitivity analyses including a two-year lag analysis to avoid reverse causation and a secondary analysis where we excluded participants with self-reported hypertension at the baseline. For further sensitivity analysis, hypertension was re-defined as self-reported hypertension or an elevated measured blood pressure (systolic pressure ≥ 140 mmHg or diastolic pressure ≥ 90 mmHg)^17^ to avoid a possible misclassification of hypertension diagnosis (n = 17,835). Crude estimates of HR were calculated to assess the influence of adjustment. Two-tailed p-values < 0.05 were considered statistically significant. All analyses were performed using the statistical software package SAS 9.4 (SAS Institute, Cary, NC, USA).

### Ethics statement

The study protocol and its procedures were approved by the Institutional Review Board (IRB) of the Seoul National University Hospital in Seoul, Korea (IRB number E-2009-117-1159) and the Korea National Institute of Health (IRB number 2014-08-02-3C-A). Written informed consent was obtained from all participants. The approved research was performed in accordance with relevant guidelines and regulations established by the Ethics Committee of the Korean National Institute of Health and the Helsinki Declaration of the World Medical Association.

## Results

Among the 73,031 women included in the HEXA-G study, 858 cases of breast cancer were identified over a follow-up period of 671,908 person-years (median 9.1 years). At baseline, participants who have SBP equal or over 130 mmHg and DBP threshold equal to or over 85 mmHg tended to be older, had higher BMI, and were more likely to have self-reported hypertension and family history of hypertension. Postmenopausal participants were found to have higher SBP and DBP values than premenopausal women (Table 1). Baseline characteristics statistically differed between menopausal status groups. Postmenopausal participants exhibited higher SBP and DBP values, a higher average BMI but higher proportion of regular physical activity, earlier age of menarche, older age of first pregnancy, and self-reported hypertension than premenopausal women. No premenopausal women were under hormone replacement treatment (Supplementary Table 1).

**Table 1:** Baseline characteristics of the HEXA-G study population by SBP and DBP binary categorization (N = 73,031).

Characteristics	Systolic blood pressure (mmHg)				*p* value^a^	Diastolic blood pressure (mmHg)				*p* value^a^
	< 130		≥ 130			< 85		≥ 85		
	N	%	N	%		N	%	N	%	
Number of participants	51,956	71.14	21,075	28.86		62,693	85.84	10,338	14.16	
Age (years, mean ± SD)	50.98	7.54	55.13	7.48	< 0.01	51.79	7.75	54.44	7.38	< 0.01
BMI (kg/m^2^, mean ± SD)	23.19	2.77	24.62	3.08	< 0.01	23.41	2.85	24.78	3.17	< 0.01
Education					< 0.01					< 0.01
≤ Middle school	16,049	61.84	9903	38.16		21,315	82.13	4637	17.87	
High School or College	23,818	73.64	8525	26.36		27,979	86.51	4364	13.49	
Bachelor or higher	12,089	82.04	2647	17.96		13,399	90.93	1337	9.07	
Smoking status					< 0.01					0.09
Never	50,162	70.92	20,566	29.08		60,642	85.74	10,086	14.26	
Ever	1794	77.90	509	22.10		2051	89.06	252	10.94	
Alcohol drinking status					< 0.01					< 0.01
Never	34,099	69.75	14,786	30.25		41,810	85.53	7075	14.47	
Ever	17,857	73.95	6289	26.05		20,883	86.49	3263	13.51	
Physical activity	26,564	70.92	10,893	29.08	0.17	32,131	85.78	5326	14.22	0.62
Familiar History of breast cancer	245	85.07	43	14.93	0.71	245	85.07	43	14.93	0.71
Age at menarche					< 0.01					< 0.01
< 9 to 14 years	18,346	66.79	9121	33.21		23,108	84.13	4359	15.87	
15 years	12,707	71.17	5148	28.83		15,288	85.62	2567	14.38	
16 or older	20,903	75.44	6806	24.56		24,297	87.69	3412	12.31	
Age at first pregnancy					< 0.01					< 0.01
No pregnancy*	2,032	77.56	588	22.44		2308	88.09	312	11.91	
< 25 years	29,023	73.67	10,374	26.33		34,310	87.09	5087	12.91	
≥ 25 years	20,901	67.39	10,113	32.61		26,075	84.07	4939	15.93	
Menopausal status					< 0.01					< 0.01
Pre-menopausal	24,314	80.69	5817	19.31		26,966	89.50	3165	10.5	
Post-menopausal	27,642	64.43	15,258	35.57		35,727	83.28	7173	16.72	
Hormone replaced treatment use					< 0.01					< 0.01
Never	44,396	71.77	17,461	28.23		53,137	85.90	8720	14.10	
Former use	5478	66.01	2821	33.99		7048	84.93	1251	15.07	
Current use	2082	72.42	793	27.58		2,508	87.23	367	12.77	
Self-reported hypertension	5317	43.23	6981	56.77	< 0.01	8675	70.54	3623	29.46	< 0.01
Family history of hypertension	15,809	66.02	8137	33.98	< 0.01	19,757	82.51	4189	17.49	< 0.01

*HEXA-G* Health Examinees Study-Gem, *SBP* systolic blood pressure, *DBP* diastolic blood pressure, *BMI* body mass index, *SD* standard deviation.

Variable distributions are reported as n(%) unless otherwise specified.

^a^Student's t-test for continuous variables; Chi-square test for categorical variables.

### Breast cancer risk and menopausal status

There was no overall evidence for association of BP absolute values with BC (SBP, HR 1.02, 95% CI 0.98–1.08; DBP, HR 1.05, 95% CI 0.97–1.13; DBP) for 10 mmHg increase. In category-based analysis, we did not find an association of SBP with the BC risk (Supplementary Table 1). Conversely, we found 40% higher risk of BC for all women in the 85 to 89 mmHg DBP group compared with the lowest DBP group (HR 1.40, 95% CI 1.09–1.79), with a non-significant p-trend (0.07) among all participants. The risk increased up to 73% in postmenopausal women (HR 1.73, 95% CI 1.28–2.33) in the DBP 85 to 89 mm Hg group (Table 2). In the binary analysis, DBP above 85 mm Hg was associated with BC risk (HR 1.40, 95% CI 1.11–2.79) (Supplementary Table 2). No association was found in the premenopausal group (HR 0.96, 95% CI 0.61–1.52) (Table 2). In the analysis with self-reported hypertension as the principal predictor, no association was found between hypertension and BC risk (HR 1.14, 95% CI 0.94–1.38) in the entire population of women or when stratifying by menopausal status (premenopausal: HR 1.06; 95% CI 0.72–1.56; postmenopausal: HR 1.16; 95% CI 0.92–1.45).

**Table 2:** Hazard ratios and 95% confidence intervals of breast cancer risk according to International Society of Hypertension classification of diastolic blood pressure (mmHg).

Variables (cases/participants)	Diastolic blood pressure (mmHg)				p for trend^a^
	< 85	85–89	90–100	≥ 100	
**All women (858/73,031)**					
Number of participants	62,693	4655	4748	935	
Breast cancer cases	716	71	60	11	
Person-years	575,653	42,761	44,659	8835	
HR (95% CI) model 1	Ref	1.38 (1.08–1.76)	1.13 (0.87–1.47)	1.03 (0.57–1.87)	0.06
HR (95% CI) model 2	Ref	1.41 (1.11–1.81)	1.15 (0.88–1.50)	1.05 (0.58–1.91)	0.04
HR (95% CI) model 3	Ref	1.40 (1.09–1.79)	1.12 (0.86–1.47)	1.03 (0.56–1.87)	0.07
**Pre-menopausal (433/30,131)**					
Number of participants	26,966	1483	1375	307	
Breast cancer cases	387	20	22	NR^b^	
Person-years	250,144	13,589	12,991	2894	
HR (95% CI) model 1	Ref	0.95 (0.61–1.49)	1.10 (0.71–1.69)	0.90 (0.33–2.40)	0.96
HR (95% CI) model 2	Ref	0.97 (0.62–1.52)	1.12 (0.72–1.72)	0.92 (0.34–2.46)	0.87
HR (95% CI) model 3	Ref	0..96 (0.61–1.52)	1.10 (0.71–1.72)	0.91 (0.34–2.44)	0.92
**Postmenopausal (425/42,900)**					
Number of participants	35,727	3172	3373	628	
Breast cancer cases	329	51	38	7	
Person-years	325,509	29,173	31,668	5941	
HR (95% CI) model 1	Ref	1.71 (1.28–2.30)	1.18 (0.84–1.64)	1.16 (0.55–2.45)	0.01
HR (95% CI) model 2	Ref	1.75 (1.30–2.36)	1.18 (0.84–1.66)	1.18 (0.56–2.49)	0.01
HR (95% CI) model 3	Ref	1.73 (1.28–2.33)	1.16 (0.82–1.63)	1.15 (0.54–2.43)	0.02

Model 1: unadjusted.

Model 2: adjusted for family history of breast cancer, body mass index, parity, age at birth of a first child, age at menopause, breastfeeding, physical activity, alcohol consumption, smoking status. In postmenopausal additionally adjusted by hormone replaced therapy use.

Model 3: adjusted for model 2 variables and self-reported history of hypertension.

^a^p-trend values were calculated with linear-by-linear association tests.

^b^Frequencies less than 5 are not reported.

### Interaction with blood pressure-related variables

In the stratification analysis, the positive association between DBP 85 to 89 mmHg and BC risk was retained in the group of women with BMI equal to or higher than 25 kg/m^2^, no regular physical activity, family history of hypertension, and no history of cardiovascular disease or diabetes. However, the interaction term and p-trend were not significant (Table 3). No differences by subgroup were found in the SBP analysis (Supplementary Table 3).

**Table 3:** Subgroup and sensitivity analysis of breast cancer risk according to diastolic blood pressure categories.

Variables	Participants	BC cases	Diastolic blood pressure (mmHg)				*p *for interaction^b^	*p* for trend
			< 85	85–89	90–100	≥ 100		
				HR (95% CI)^a^	HR (95% CI)^a^	HR (95% CI)^a^		
**Body mass index (Kg/m**^**2**^**)**								
< 23.0	33,095	402	Ref	1.41 (0.93–2.14)	0.98 (0.60–1.60)	0.91 (0.29–2.86)	0.66	0.48
23.0–25.0	19,418	209	Ref	0.99 (0.55–1.79)	1.33 (0.79–2.24)	0.95 (0.23–3.84)		0.50
≥ 25.0	20,518	247		1.65 (1.14–2.37)	1.15 (0.76–1.73)	1.13 (0.50–2.56)		0.11
**Age at baseline (years)**								
40–49	27,713	390	Ref	1.26 (0.81–1.97)	1.24 (0.76–2.01)	0.87 (0.28–2.74)	0.46	0.34
50–59	30,348	329	Ref	1.42 (0.98–2.06)	1.13 (0.76–1.70)	1.09 (0.45–2.64)		0.18
60–69	14,970	139	Ref	1.65 (0.99–2.73)	1.08 (0.62–1.86)	1.28 (0.40–4.05)		0.25
**Physical activity**								
Yes	37,457	442	Ref	1.30 (0.91–1.86)	1.24 (0.87–1.77)	1.10 (0.49–2.47)	0.35	0.14
No	35,574	416	Ref	1.49 (1.06–2.11)	0.99 (0.66–1.50)	0.96 (0.39–2.32)		0.31
**History of cardiovascular disease**								
Yes	1854	18	Ref	1.99 (0.44–9.05)	2.41 (0.65–8.88)		0.68	0.27
No	71,177	840	Ref	1.38 (1.08–1.78)	1.09 (0.83–1.43)	1.03 (0.57–1.87)		0.09
**History of diabetes mellitus**								
Yes	3660	31	Ref	2.30 (0.86–6.19)	1.49 (0.50–4.46)		0.53	0.37
No	69,371	827	Ref	1.36 (1.06–1.76)	1.10 (0.84–1.46)	1.08 (0.59–1.96)		0.09
**Family history of hypertension**								
Yes	23,946	298	Ref	1.50 (1.03–2.20)	0.94 (0.61–1.46)	0.98 (0.40–2.39)	0.54	0.07
No	49,085	560	Ref	1.33 (0.96–1.85)	1.27 (0.90–1.79)	1.06 (0.47–2.38)		0.48
**Sensitivity analysis**								
Two years lag-time	72,899	726	Ref	1.44 (1.10–1.87)	1.14 (0.86–1.53)	0.77 (0.37–1.64)		0.13
Excluded self-reported HTN	60,733	715	Ref	1.42 (1.06–1.90)	1.34 (0.98–1.84)	0.82 (0.34–1.97)		0.03

*HR* hazard ratio, *CI* confidence intervals, *HTN* hypertension.

^a^Adjusted for family history of breast cancer, parity, age at birth of the first child, age at menarche, breastfeeding, hormone replaced therapy use, physical activity, alcohol consumption, smoking status, self-reported history of hypertension.

^b^Interaction was calculated using Wald test of cross-product terms.

### Sensitivity analysis

Adjusted analysis of combined self-reported and measured blood pressure showed an association with BC risk (HR 1.19; 95% CI 1.01–1.41) regardless menopausal status (premenopausal: HR 1.20; 95% CI 0.91–1.59; postmenopausal: HR 1.17; 95% CI 0.95–1.45). Analyses excluding individuals who presented BC in the first two years of follow-up showed BP associations with BC risk that were similar to those observed overall (Table 3). DBP within the 85–89 mmHg category was related to 44% increased BC risk in all women (HR 1.44; 95% CI 1.10–1.87), and 80% increased BC risk in postmenopausal women (HR 1.82; 95% CI 1.31–2.47). Additionally, in separate analyses excluding all patients with self-reported hypertension, similar results were found for SBP and DBP (Supplementary Fig. 1).

## Discussion

We examined the relationship between SBP and DBP with the risk of BC in a large population-based cohort study. We found that DBP was associated with an increased risk of BC for postmenopausal women, independently of previous diagnoses of hypertension. We observed a strong positive association of DBP values over 85 mmHg with BC, particularly in the range of 85 to 89 mmHg among postmenopausal women. This finding agrees with a study where DBP over 85 mmHg was associated with BC risk in a population of American postmenopausal women^7^. Similarly, a recent study using the European Prospective Investigation into Cancer and Nutrition (EPIC) cohort reported a positive association of both SBP and DBP categorized by the American Hypertension Association (AHA) and the European Society of Hypertension with BC risk in postmenopausal but not in premenopausal women^6^. In contrast to our results, this European study reported a significant increase of 3% BC risk per 10 mmHg increase in SBP and DBP and a positive dose response among BP categories. These differences might be explained by population characteristics and genetic heterogeneity between population studies^18^. A study from Norway, Austria and Sweden reported an increased BC risk for the highest quintile values of SBP and DBP for all women^8^. Conversely, a prospective study from Australia found no association between BP measurements and BC risk^9^. However, in a secondary analysis, DBP between baseline and the second follow-up wave were associated with triple negative breast cancer risk. While the SBP associations in previous studies are conflicting, our study did not find an association between SBP and BC risk, presumably due to dissimilarities in population characteristics and BP cutoff ascertainment. Furthermore, similar to previous pooled cohort studies^4,5^, we found that hypertension, defined as self-reported combined with objectively measured blood pressure, is positive associated with BC risk.

These epidemiological findings must be put in context of the physiopathology of hypertension and breast cancer. The physiological link between high BP and cancer risk is still unclear. One main issue about BP parameters is that while age is positively correlated with SBP increase, DBP is not as straightforward. SBP increases continuously with age, whereas DBP increases until the fifth decade and then slowly decreases from the sixth decade due to vascular stiffness as part of the normal aging process^19^. DBP has two major components: peripheral vascular resistance (PVR) and artery compliance^20,21^. PVR is the total resistance to blood flow across the vascular system determined by the small arterioles. Artery compliance refers to the distensibility of the blood vessels due to blood volume, a term which is related to the elasticity of blood vessels^22^. If PVR and artery compliance increase, then DBP would increase. However, aging reduces artery compliance and/or increases artery stiffness, decreasing DBP^19^. Even though there is evidence suggesting that both peripheral (muscular) and central (elastic) arteries in hypertensive individuals are stiffer compared to normotensive individuals, in individuals with isolated hypertension, where the SBP is increased with a normal DBP value, the stiffness is increased in large aortic but not peripheral arteries^23,24^. Hence, increased DBP values might negatively affect blood flow fluctuations in the peripheral vessels affecting organs like the breast.

In addition to age, other factors increase BP, including decreased baroreceptor and chemoreceptor sensitivity, increased responsiveness to sympathetic nervous system stimuli, altered sodium metabolism, and altered renin-angiotensin metabolism. Additionally, after menopause, women lose estrogen-vascular protection^25^. The decrease in endogenous estrogens, mediated by estrogen receptors (ERs), leads to endothelial vasoconstriction, increasing peripheral vascular resistance^26–29^. Therefore, lower estrogen levels in blood might also contribute to lower arterial compliance and increased risk of high BP in postmenopausal women^30–32^.

Thus, slight alterations in the arterial lumen, either functional or structural, result in significant changes in arterial resistance. Moreover, menopause has been shown to accelerate age-related rises in sympathetic nerve activity (SNA), related to impaired central modulation of baroreflex function and direct inhibitory influence of estrogen on SNA^33^. The increased activity of SNA increases the PVD, increasing BP. However, whether menopause or estrogen have a larger effect on DBP than SBP has not been reported to the best of our knowledge. It is possible that with altered central autonomic regulation coupled with enhanced vascular adrenergic sensitivity may be responsible for elevated DBP and exaggerated pressor responses to exercise and mental stress in some postmenopausal women^33,34^.

**Figure 2:**
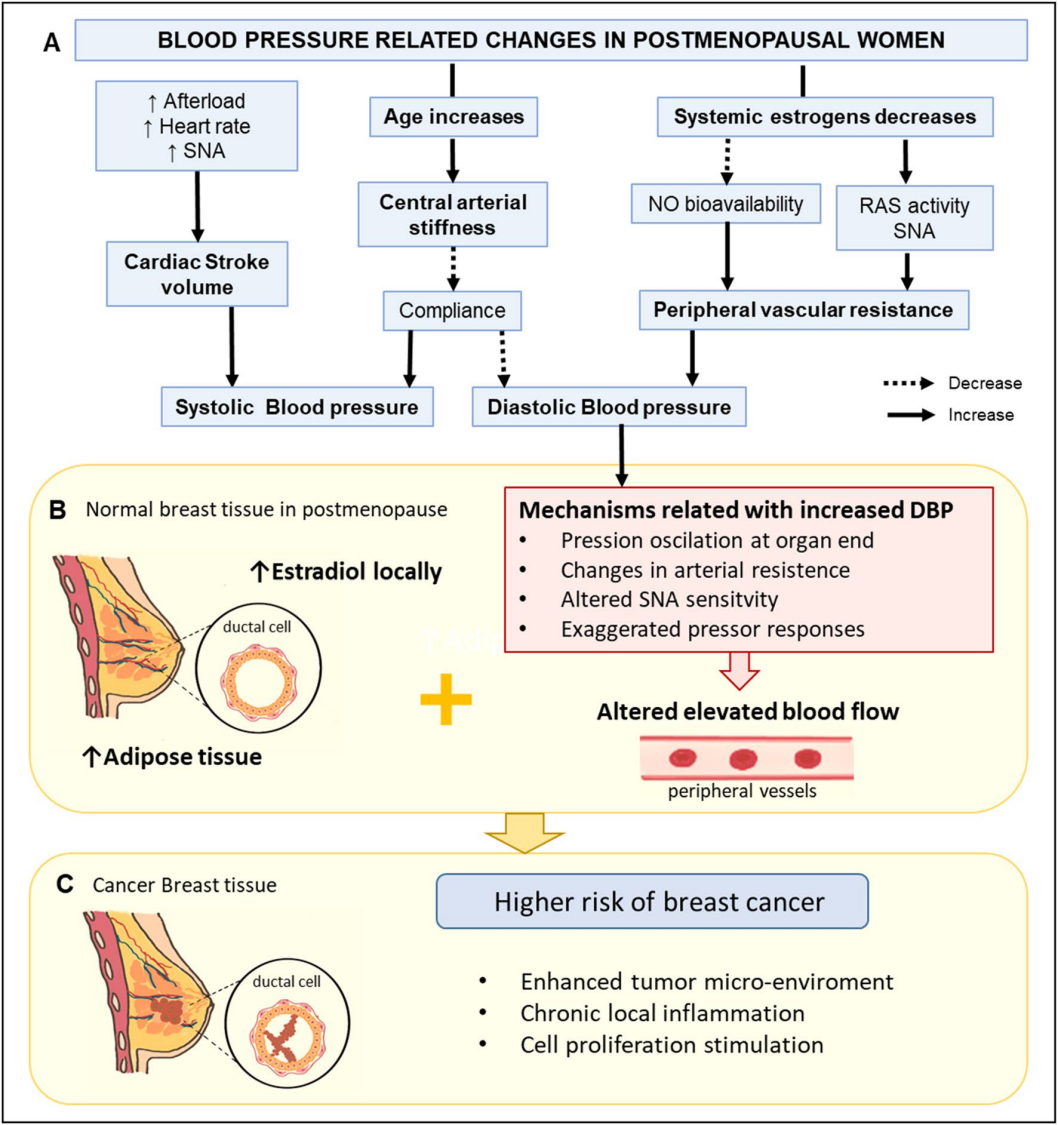
Putative mechanism related to DBP and increased risk of breast cancer in postmenopausal women. (**A**) Physiological responses for an increase in age and decrease in systemic estrogens and their influence on the SBP and DBP. (**B**) Hypothetical mechanisms related to increased DBP and altered blood flow and its association with increased estrogen production in breast cancer tissue. (**C**) Local estradiol and altered blood flood in breast tissue could enhance tumor microenvironment, local inflammation, and cell proliferation stimulation, increasing breast cancer risk. *NO* nitric oxide, *RAS* renin-angiotensin system, *SNA* sympathetic nerve activity, *SBP* systolic blood pressure, *DBP* diastolic blood pressure. The figure was generated using Microsoft PowerPoint (2016) and Sketchbook (iOS version 5.2.2).

In postmenopausal women, local adipose tissue increases and stroma tissue diminishes in the breast. Adipose cells in breast tissue produce estradiol locally from circulating precursors^35^. Therefore, estradiol levels are higher in breast tissue than in the bloodstream, especially in postmenopausal women^36,37^. Genotoxic metabolites from estradiol contribute to BC development^38^. Hence, high local estrogen production and altered blood flow in breast tissue may contribute to chronic local inflammation, cell proliferation stimulation, and tumor micro-environment enhancement^39^. Therefore, we propose that increased DBP might not be a causal risk factor for BC, but it might be an important contributor to BC development in postmenopausal women. In this context, postmenopausal women with less-aged vessels in the breast area due to local increased estrogen production accompanied by altered high blood flow in the peripheral vessels are more likely to experience BC (Fig. 2). Although, SBP has been associated with breast cancer in other studies^6^, SBP is more related to the cardiac stroke volume than the PVR. Therefore, as PVR increases, SBP will also increase but not as much as DBP^40,41^. On contrary, in premenopausal women, no association between BP and BC risk might be partially explained by the estrogen’s vascular protective factors in this period^26,27,42^.

High BP, a consequence of the interaction of genetic and environmental factors, causes oxidative stress resulting in smooth muscle hypertrophy and spasm, endothelial dysfunction, subendothelial low density lipoprotein deposition and oxidation in the vascular walls^43,44^. Similarly, breast cancer may be attributed to oxidative stress in old age where protein damage, DNA damage, and lipid peroxidation may increase the BC risk independently^45,46^. Proposed biological mechanisms for explaining the link between high BP and BC include chronic inflammation process, modification in apoptosis activation, and disequilibrium in the renin-angiotensin system^47–52^, which might be related to BP components, but further research is needed. Recently an in-vitro study showed that G protein coupled receptor kinase 4 (GRK4), a regulator of renal sodium excretion, functions as an independent proliferation promotor in BC cells^53^. Furthermore, evidence suggests that controlling BP may reduce the risk of BC^54^. Although the evidence is still contradictory and associated to specific antihypertensive drugs^54,55^, the positive association between hypertension and BC risk disappeared when treated vs. untreated hypertension was evaluated in a large European cohort study^6^.

Being overweight or obese has been related to breast cancer risk^56^ and hypertension^57^. We analyzed the association of DBP and breast cancer risk by BMI subgroups and found no interaction, which means that the effect of DBP on breast cancer risk is independent of women BMI.

This study has some limitations. First, there is a lack of information on changes over time for BP measurements and some potential confounding self-reported variables, which may result in misclassification bias. Although our models were adjusted for the use of HRT, we lack information on the type and duration of HRT which may affect the association in postmenopausal women. Furthermore, we lack information on the type of hypertension treatment or antihypertensive drug used in hypertensive individuals which may have reduced the strength of hypertension associated with breast cancer. Also self-reported hypertension might be misleading due to measurement error or recall bias. Additionally, only one measured hypertensive value is not enough for hypertension diagnosis. Therefore, combining both methods might improve the underestimation of a hypertension diagnosis in the “healthy” population. Hence, we included self-reported hypertension diagnosis as a confounding variable in our third model, aiming to control both the antihypertensive treatment and the length of the hypertension diagnosis^58^. Also, the secondary analyses excluding hypertensive individuals showed that previous hypertension diagnosis did not affect the association of normal-high DBP values and BC incidence.

One of the challenges of working with BP measurements is classification. The criteria used to define BP cut-offs have been changing over the last few decades depending upon different approaches. We selected the cut-offs of the 2020 International Society Hypertension guidelines as our categorization criteria due to their recent publication, worldwide use, and tailored use in low and high resources settings^15^. Additionally, as this population-based cohort includes mainly “healthy participants”, we analyzed BP using binary cut-offs at the normal category for DBP and SBP to increase the statistical power for the higher BP groups.

Despite the limitations, the strengths of our study include its prospective study design in a large cohort, the availability of objectively measured SBP and DBP by trained staff and the high accuracy of BC diagnoses retrieved from the KCCR. This study is the first in our knowledge to examine the association of BP measurements and BC risk in an Asian large-based population cohort. Moreover, the number of participants and BC cases were similar among premenopausal and postmenopausal women, which allows us to draw conclusions. However, we cannot exclude the possible misclassification of perimenopausal women. Additionally, detailed information of established risk factors for breast cancer such as reproductive factors, family history of BC, smoking, and lifestyle risk factors was available, enabling adjustment for potential confounders.

In summary, this study suggests that normal-high DBP values are associated with increased BC risk in postmenopausal women. We propose that mildly elevated DBP values are associated with an increased probability of BC, but they are not necessarily causal factors. Still, DBP could be used for the risk calculation of BC occurrence. Our observations suggest the importance of DBP to estimate BC risk, particularly in postmenopausal women. This epidemiological association still requires further research in larger samples and, ultimately, potential mechanisms involved need to be elucidated.

## Supplementary Information

Supplementary Information.

## Data Availability

Raw data for this study came from the Health Examinees (HEXA) study as part of the Korean Genome and Epidemiology Study (KoGES), conducted by Korea Disease Control and Prevention Agency (KDCA; formerly Korea Centers for Disease Control and Prevention), Republic of Korea. The Korea Central Cancer Registry (KCCR) data is provisioned by the KDCA in cooperation with the National Cancer Center of Korea as a part of the KoGES. The dataset used for the analysis in this study is maintained and managed by the Division of Population Health Research at the National Institute of Health, which is a part of the Korea Disease Control and Prevention Agency. The Health Examinees Study dataset has been merged with the cancer registry data provided by National Cancer Center of Korea in a collaborative agreement. The data generated in this study are not publicly available due to inclusion of personal data that may potentially be sensitive to the patients, even though researchers are provided with an anonymized dataset that excludes resident registration numbers. Derived data supporting the findings of this study are available from the corresponding author upon request.
