# Supplementary material for: Mildly elevated diastolic blood pressure increases subsequent risk of breast cancer in postmenopausal women in the Health Examinees-Gem study
Source: Sci Rep. 2022 Sep 26;12:15995. doi: 10.1038/s41598-022-19705-4 (PMC9512811; doi:10.1038/s41598-022-19705-4)
Supplement: Supplementary file 1 — Supplementary Information. [file 41598_2022_19705_MOESM1_ESM.pdf]

Mildly elevated diastolic blood pressure increases subsequent risk of breast cancer in postmenopausal women in the Health Examinees-Gem study

Katherine De la Torre [1,2], Woo-Kyoung Shin [1,7], Dan Huang [1,7], Hwi-Won Lee [1,2], Aesun Shin [1,3,7], Jong-koo Lee [4], Hae-Young Lee [5,6], Daehee Kang [1,7*]

[1] Department of Preventive Medicine, Seoul National University College of Medicine, Seoul, 03080, Korea

[2] Department of Biomedical Sciences, Seoul National University Graduate School, Seoul, 03080, Korea

[3] Cancer Research Institute, Seoul National University, Seoul, 03080, Korea

[4] Department of Family Medicine, Seoul National University Hospital, Seoul, 03080, Korea

[5] Department of Internal Medicine, Division of Cardiology, Seoul National University Hospital, Seoul, 03080, Korea

[6] Department of Internal Medicine, Seoul National University College of Medicine, Seoul, 03080, Korea

[7] Integrated Major in Innovative Medical Science, Seoul National University Graduate School, Seoul, 03080, Korea

*Corresponding author: dhkang@snu.ac.kr

Supplementary Table 1. Baseline characteristics of the HEXA-G study population by menopausal status

	Menopausal Status				*p* value[a]
	Premenopausal		Postmenopausal		
	N	%	N	%	
Number of participants	30,131	41.26	42,900	58.74	
Person-years	279,617		392,291		
Follow-up time, median years	9.22		9.13		
Breast cancer cases	425	49.53	433	50.47	<0.01
SBP (mmHg, mean ± SD)	116.9	14.11	123.27	14.96	<0.01
DBP (mmHg, mean ± SD)	72.88	9.47	75.95	9.41	<0.01
Age (years, mean ± SD)	45.50	4.19	57.12	5.83	<0.01
Age at baseline, years					<0.01
40-49	24,122	87.04	3,591	12.96	
50 a 59	6,009	19.96	24,390	80.37	
>60	NA	NA	14,919	99.66	
BMI (kg/m^2, mean ± SD)	23.07	2.90	23.98	2.90	<0.01
Education					<0.01
≤Middle school	4,646	17.9	21,306	82.10	
High School or College	16,051	49.63	16,292	50.37	
Bachelor or higher	9,431	64.02	5,302	35.98	
Smoking status					<0.01
Never	29,049	41.07	41,679	58.93	
Ever	1,082	46.98	1,221	53.02	
Alcohol drinking status					<0.01
Never	17,257	35.3	31,628	64.70	
Ever	12,874	53.32	11,272	46.68	
Physical activity					<0.01
Yes	14,778	39.45	22,679	60.55	
No	15,353	43.16	20,221	56.84	
Familiar history of breast cancer					0.07
Yes	134	46.53	154	53.47	
No	29,997	41.24	42,746	58.76	
Age at menarche					<0.01
<9 to 14 years	7,065	25.72	20,402	74.28	
15 years	7,661	42.91	10,194	57.09	
16 or more	15,405	55.6	12,304	44.4	
Age at first pregnancy					<0.01
No pregnancy*	1,543	58.89	1,077	41.11	
<25 years	18,686	47.43	20,711	52.57	
>=25 years	9,902	31.93	21,112	68.07	
Breastfeeding					<0.01
Yes	22,450	37.63	37,204	62.37	
No	7,681	57.42	5,696	42.58	
Hormone replaced treatment use					<0.01
Never	30,131	48.71	31,726	51.29	
Former use	NA	NA	8,299	100	
Current use	NA	NA	2,875	100	
Self-reported hypertension					<0.01
No	27,358	46.88	31,002	53.12	
Yes	2,773	18.90	11,898	81.10	
Family history of hypertension					<0.01
No	19,616	39.96	29,469	60.04	
Yes	10,515	43.91	13,431	56.09	

HEXA-G, Health Examinees Study-Gem; SBP, systolic blood pressure; DBP, diastolic blood pressure; BMI, body mass index; SD, standard deviation; NA, no applicable

Variable distributions are reported as n(%) unless otherwise specified.

[a] Student's t-test for continuous variables; Chi-square test for categorical variables

Supplementary Table 2. Hazard ratios and 95% confidence intervals of breast cancer risk according to International Society of Hypertension classification of systolic blood pressure (mmHg)

	Systolic Blood Pressure (mm Hg)				
**Variables (cases/participants)**	**<130**	**130 - 139**	**140 - 159**	**≥160**	**p for trend[a]**
**All women (858/73,031)**					
Number of Participants	51,956	13,309	6,702	1,064	
Breast cancer cases	623	141	81	13	
Person-years	479,035	121,483	61,421	9,969	
HR (95% CI) Model 1	ref	0.93 (0.78 - 1.12)	1.08 (0.87 - 1.37)	1.06 (0.61 - 1.84)	0.83
HR (95% CI) Model 2	ref	0.96 (0.80 - 1.16)	1.12 (0.88 - 1.42)	1.10 (0.63 - 1.92)	0.57
HR (95% CI) Model 3	ref	0.95 (0.78 - 1.14)	1.09 (0.85 - 1.39)	1.06 (0.61 - 1.85)	0.78
**Premenopausal (433/30,131)**					
Number of Participants	24,314	3,920	1,601	296	
Breast cancer cases	354	44	32	NR[b]	
Person-years	226,155	35,995	14,675	2,792	
HR (95% CI) Model 1	ref	0.78 (0.57 - 1.07)	1.39 (0.97 - 2.00)	0.69 (0.22 - 2.17)	0.96
HR (95% CI) Model 2	ref	0.79 (0.58 - 1.09)	1.44 (0.99 - 2.08)	0.72 (0.23 - 2.25)	0.82
HR (95% CI) Model 3	ref	0.79 (0.57 - 1.09)	1.43 (0.98 - 2.09)	0.71 (0.23 - 2.23)	0.87
**Postmenopausal (425/42,900)**					
Number of Participants	27,642	9,389	5,101	768	
Breast cancer cases	269	97	49	10	
Person-years	252,880	85,488	46,746	7,177	
HR (95% CI) Model 1	ref	1.05 (0.83 - 1.33)	0.97 (0.71 - 1.32)	1.29 (0.68 - 2.42)	0.70
HR (95% CI) Model 2	ref	1.07 (0.84 - 1.35)	0.99 (0.72 - 2.08)	1.34 (0.71 - 2.54)	0.58
HR (95% CI) Model 3	ref	1.05 (0.82 - 1.33)	0.95 (0.69 - 1.30)	1.28 (0.68 - 2.43)	0.78

Model 1: unadjusted
Model 2: adjusted for family history of breast cancer, body mass index, parity, age at birth of a first child, age at menopause, breastfeeding, physical activity, alcohol consumption, smoking status. In postmenopausal additionally adjusted by hormone replaced therapy use.
Model 3: adjusted for model 2 variables and self-reported history of hypertension.
[a] p-trend values were calculated with linear-by-linear association tests.
[b] Frequencies less than 5 are not reported

Supplementary Table 3. Hazard ratios and 95% confidence intervals of breast cancer risk according SBP and DBP dichotomous stratification

	Systolic Blood Pressure (mm Hg)		Diastolic Blood Pressure (mm Hg)	
**Variables (cases/participants)**	**<130**	**≥130**	**<85**	**≥ 85**
**All women (858/73031)**				
Number of Participants	51,956	21,075	62,693	10,338
Breast cancer cases	623	235	716	142
Person- years	479,035	192,873	575,653	96,255
HR (95% CI) Model 1	ref	0.99 (0.85 - 1.15)	ref	1.23 (1.03 - 1.48)
HR (95% CI) Model 2	ref	1.02 (0.87 - 1.19)	ref	1.26 (1.05 - 1.51)
HR (95% CI) Model 3	ref	0.99 (0.85 - 1.17)	ref	1.23 (1.03 - 1.49)
**Premenopausal (433/30,131)**				
Number of Participants	24,314	5,817	26,966	3,165
Breast cancer cases	354	79	387	46
Person- years	226,155	53,462	250,144	29,473
HR (95% CI) Model 1	ref	0.94 (0.74 - 1.21)	ref	1.01 (0.74 - 1.37)
HR (95% CI) Model 2	ref	0.96 (0.75 - 1.24)	ref	1.03 (0.75 - 1.40)
HR (95% CI) Model 3	ref	0.95 (0.74 - 1.23)	ref	1.02 (0.74 - 1.40)
**Postmenopausal (425/42,900)**				
Number of Participants	27,642	15,258	35,727	7,173
Breast cancer cases	269	156	329	96
Person- years	252,880	139,411	325,509	66,782
HR (95% CI) Model 1	ref	1.04 (0.85 - 1.26)	ref	1.41 (1.12 - 1.77)
HR (95% CI) Model 2	ref	1.05 (0.86 - 1.29)	ref	1.43 (1.14 - 1.80)
HR (95% CI) Model 3	ref	1.03 (0.83 - 1.26)	ref	1.40 (1.11 - 1.79)

Model 1: unadjusted
Model 2: adjusted for family history of breast cancer, body mass index, parity, age at birth of a first child,age at menopause, breastfeeding, physical activity, alcohol consumption, smoking status. In postmenopausal additionally adjusted by hormone replaced therapy use.
Model 3: adjusted for model 2 variables and self-reported history of hypertension.

Supplementary Table 4. Subgroup and sensitivity analysis of breast cancer risk according to systolic blood pressure categories

Variables	Participants	BC cases	Systolic Blood Pressure (mm Hg) <130	130 - 139 HR (95% CI)[a]	140 - 159 HR (95% CI)[a]	≥160 HR (95% CI)[a]	*p for interaction*[b]	*p for trend*
**Body mass index (Kg/m²)**								
< 23.0	33,095	402	ref	1.05 (0.78 - 1.41)	0.98 (0.63 - 1.54)	1.69 (0.75 - 3.84)		0.52
23.0 - 25.0	19,418	209	ref	0.86 (0.59 - 1.27)	0.97 (0.58 - 1.62)	0.81 (0.20 - 3.32)	0.51	0.58
>= 25.0	20,518	247	ref	0.90 (0.65 - 1.24)	1.21 (0.85 - 1.73)	0.80 (0.33 - 1.96)		0.79
**Age at baseline (years)**								
40 - 49	27,713	390	ref	0.95 (0.69 - 1.32)	1.27 (0.81 - 2.01)	0.67 (0.17 - 2.71)		0.84
50 - 59	30,348	329	ref	0.89 (0.66 - 1.20)	1.37 (0.97 - 1.92)	1.19 (0.52 - 2.70)	0.88	0.3
60 - 69	14,970	139	ref	1.05 (0.72 - 1.55)	0.65 (0.38 - 1.11)	1.23 (0.49 - 3.08)		0.43
**Physical activity**								
Yes	37,457	442	ref	0.82 (0.62 - 1.08)	1.11 (0.80 - 1.54)	1.49 (0.78 - 2.82)	0.60	0.76
No	35,574	416	ref	1.09 (0.84 - 1.41)	1.06 (0.73 - 1.52)	0.54 (0.17 - 1.69)		0.99
**History of cardiovascular disease**								
Yes	1,854	18	ref	0.78 (0.22 - 2.85)	1.27 (0.34 - 4.81)		0.54	0.87
No	71,177	840	ref	0.95 (0.78 - 1.15)	1.08 (0.84 - 1.38)	1.09 (0.63 - 1.90)		0.77
**History of diabetes mellitus**								
Yes	3,660	31	ref	1.28 (0.56 - 2.83)	0.34 (0.08 - 1.51)	1.02 (0.13 - 7.94)	0.67	0.42
No	69,371	827	ref	0.92 (0.76 - 1.12)	1.15 (0.90 - 1.47)	1.07 (0.60 - 1.91)		0.65
**Family history of hypertension**								
Yes	23,946	298	ref	1.09 (0.82 - 1.47)	1.03 (0.70 - 1.52)	1.03 (0.45 - 2.36)	0.85	0.93
No	49,085	560	ref	0.86 (0.67 - 1.10)	1.15 (0.84 - 1.57)	1.08 (0.51 - 2.30)		0.73
**Sensitivity analysis**								
Two years lag -time	72,899	726	ref	0.93 (0.76 - 1.15)	1.05 (0.80 - 1.36)	0.66 (0.31 - 1.39)		0.51
Excluded self-reported HTN	60,733	715	ref	0.95 (0.76 - 1.18)	1.33 (1.01 - 1.77)	0.67 (0.25 - 1.8)		0.57

HR, hazard ratio; CI, confidence intervals; HTN, hypertension

a Adjusted by family history of breast cancer, parity, age at birth of the first child, age at menarche, breastfeeding, hormone replaced therapy use, physical activity, alcohol consumption, smoking status, self-reported history of hypertension.

b Interaction was calculated using Wald test of cross-product terms.

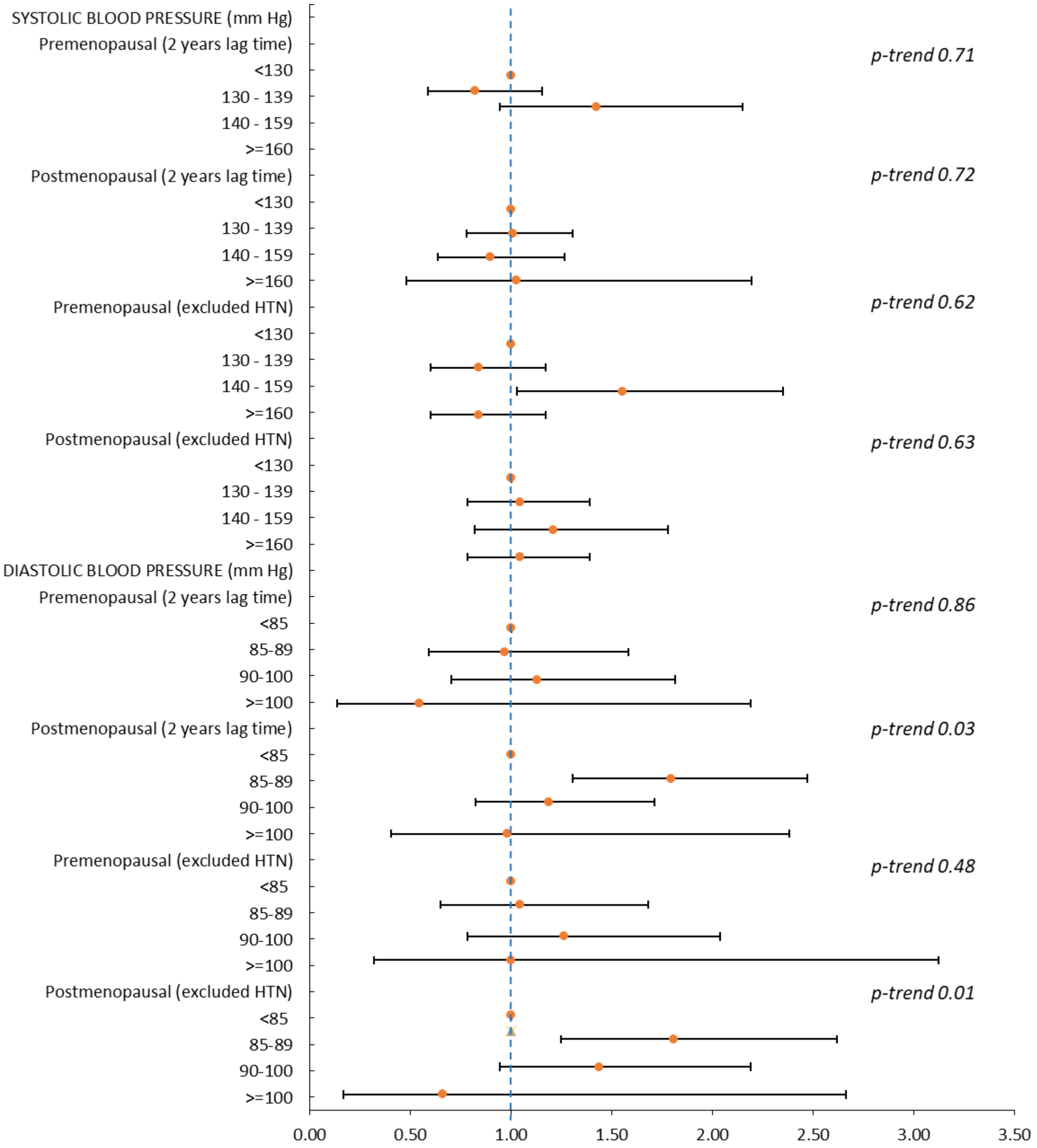

Supplementary Figure 1. Systolic and diastolic blood pressure and breast cancer risk excluding first two years of follow-up (N=72,899) and self-reported history of hypertension (N=60,733) by menopausal status
